# Supplementary material for: In situ small-angle X-ray scattering reveals strong condensation of DNA origami during silicification
Source: Nat Commun. 2022 Sep 27;13:5668. doi: 10.1038/s41467-022-33083-5 (PMC9515200; doi:10.1038/s41467-022-33083-5)
Supplement: Supplementary file 1 — Supplementary Information [file 41467_2022_33083_MOESM1_ESM.docx]

Supplementary Information

**In situ small-angle X-ray scattering reveals strong condensation of DNA origami during silicification** Martina F. Ober^1^, Anna Baptist^2^, Lea Wassermann^2^, Amelie Heuer-Jungemann^2,*^, and Bert Nickel^1,*^

^1^ Faculty of Physics and CeNS, Ludwig-Maximilians-Universität München, Geschwister-Scholl-Platz 1, 80539 Munich, Germany

^2^ Max Planck Institute of Biochemistry and CeNS, Ludwig-Maximilians-Universität München, Am Klopferspitz 18, 82152 Martinsried, Germany

Corresponding authors: *heuer-jungemann@biochem.mpg.de, nickel@lmu.de

1: DNA origami shapes by design 1

2: Custom-built sample tumbler 4

3: Small angle X-ray scattering (SAXS) analysis 5

4: Calculation of the Porod invariant 6

5: Contrast matching 7

6: Silicification of 24HBs – Parameter overview 8

7: Silicification of 4-LBs – Parameter overview 8

8: Silicification of 3-LBs – Parameter overview 9

9: In situ silicification of 24HBs monitored by SAXS – Lorentzian peaks 10

10: SAXS data of 24HBs exposed to TMAPS only for several hours 11

11: Temperature stability of ultrathin silica-coated 4-LBs 12

12: TEM images of aggregated silicified 4-LBs 13

13: In-situ silicification of 3-LBs with a square lattice design 13

14: The influence of TMAPS on the 3-LBs 15

15: Estimation of TMAPS-TEOS primary particle size 16

# **1: DNA origami shapes by design**


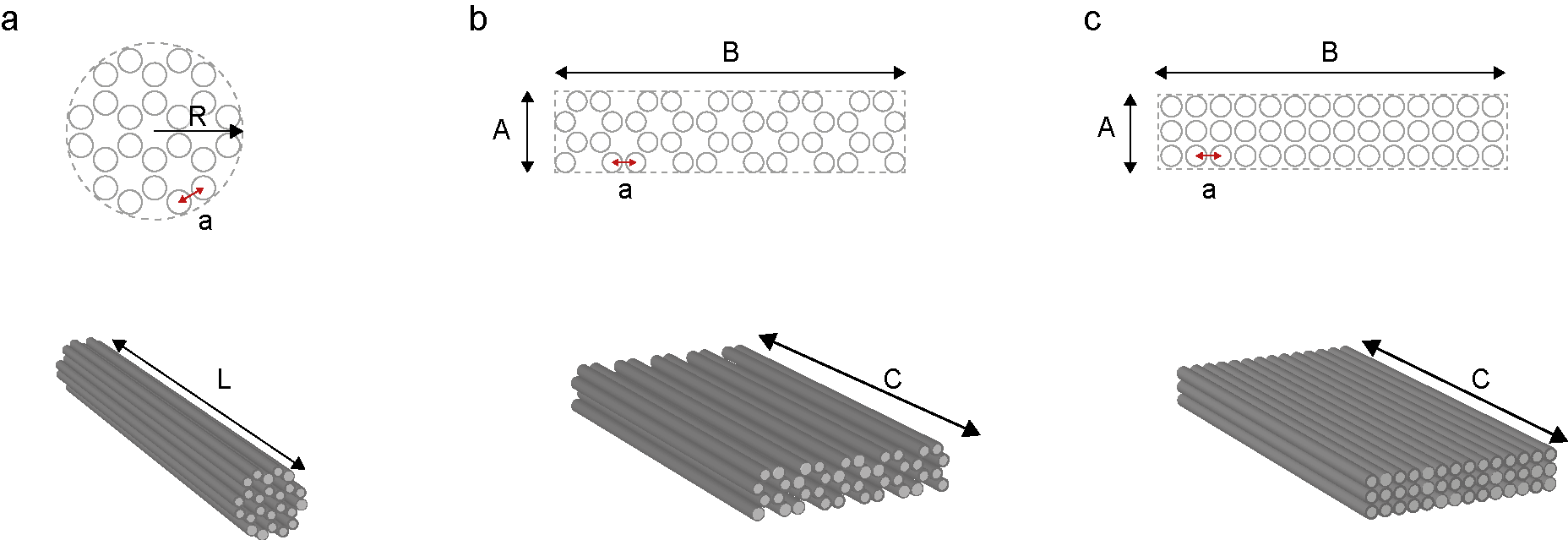


**Supplementary Figure 1** Schematic DNA origami shapes and front views of a 24-HB with a honeycomb lattice design (a), a 4-LB with a honeycomb lattice design (b), and a 3-LB with a square lattice design (c). Each solid cylinder represents a DNA double helix.

| DNA origami | A(Å) | B(Å) | C(Å) | R(Å) | L(Å) | #(helices) |
| --- | --- | --- | --- | --- | --- | --- |
| 24-HB | - | - | - | 79 | 1000 | 24 |
| 4-LB | 78 | 270 | 571 | - | - | 40 |
| 3-LB | 72 | 358 | 571 | - | - | 42 |

**Supplementary Table 1** DNA origami dimensions by design. The values are calculated in Angström assuming a base pair distance of 0.34 nm, an average interhelical distance of 2.6 nm and a radius of a DNA double helix of 1 nm.


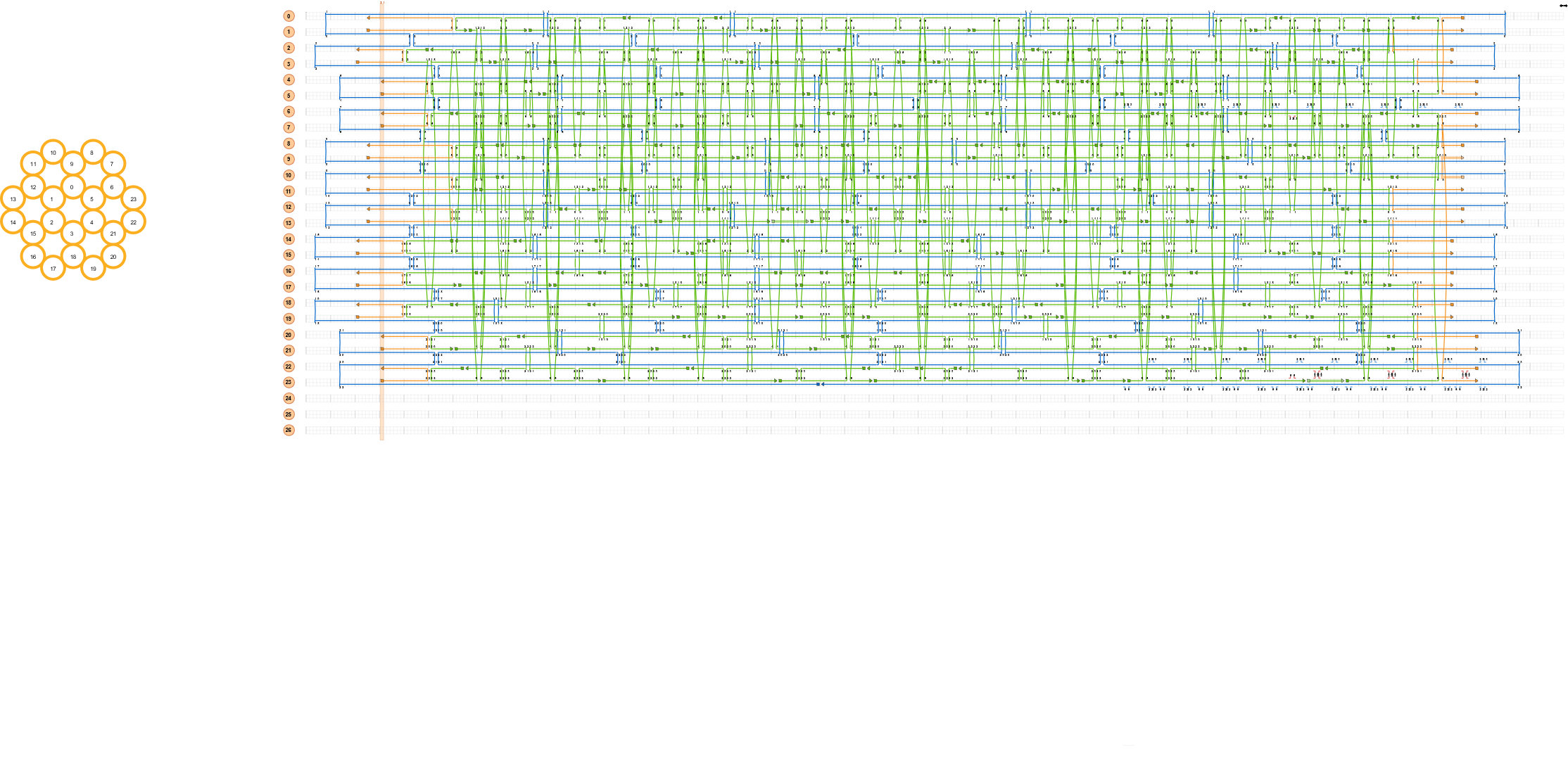


**Supplementary Figure 2** Design diagram of the 24HB using caDNAno and cross-sectional view of the helices


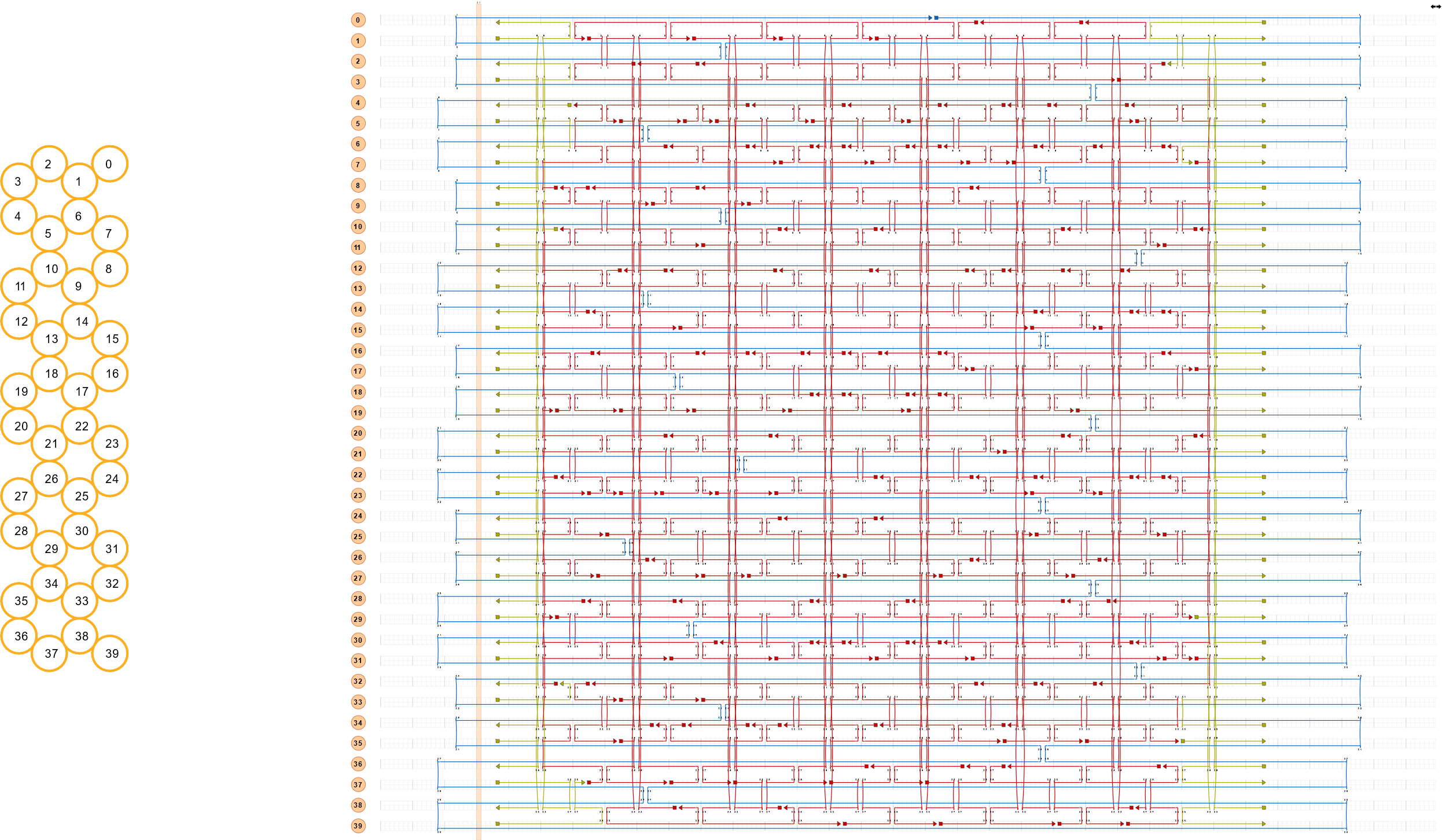


**Supplementary Figure 3** Design diagram of the 4-LB using caDNAno and cross-sectional view of the helices

# **2: Custom-built sample tumbler**


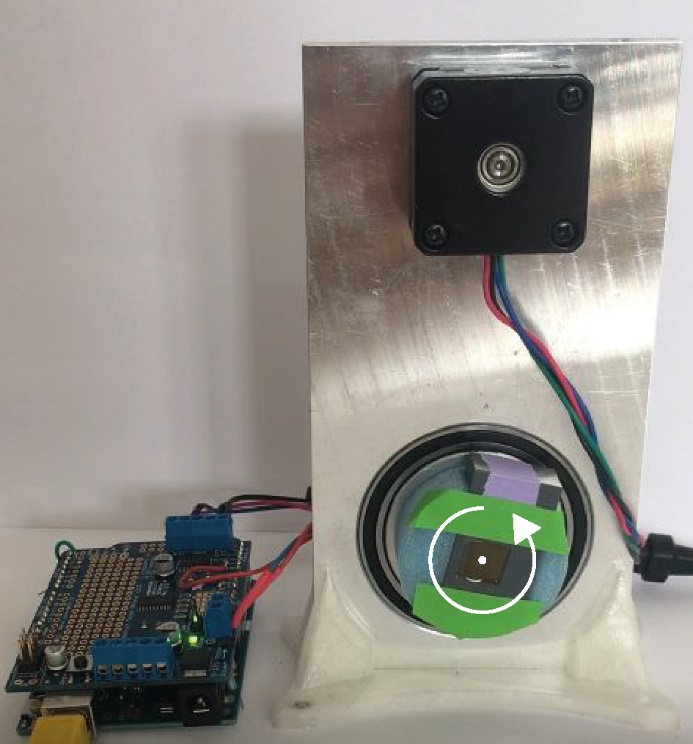


**Supplementary Figure 4** Photograph of our custom-built sample tumbler, which rotates the sample with ~ 1 round/s around the X-ray beam axis to avoid sedimentation of silicificated origami.

# **3: Small angle X-ray scattering (SAXS) analysis**

All DNA origami dimensions, as well as their inner structure, are obtained from model fits of the total scattering intensity *I(q)* to a geometrical model describing the overall origami shape with additional peaks accounting for the inner helix arrangement^1^. The scattering intensity is reported in dependence of the scattering vector
$q=\frac{4\pi}{\lambda}\sin\frac{2\theta}{2}$. Here, $\lambda$ is the X-ray wavelength and $2\theta$ is the scattering angle. To analyse the scattering data of 24HB@SiO_2_, we use a cylinder model together with a Debye background to account for free oligonucleotides, a power law to include the effects of aggregation, and Lorentzian peaks to account for the inner honeycomb lattice structure, as given in equations (3.1) - (3.5).

| $I\left( q \right)\propto\frac{s_{\text{cyl}}}{V_{\text{cyl}}}\int_{0}^{\frac{\pi}{2}} {F_{\text{cyl}}}^{2}\left( q \right)\sin\left( \alpha\right)d\alpha+s_{\text{deb}}F_{\text{deb}}\left( q \right)+s_{\text{pow}}F_{\text{pow}}\left( q \right)+s_{\text{hk}}F_{\text{lor}}\left( q \right)$ | (3.1) |
| --- | --- |
| $F_{\text{cyl}}\left( q \right)=\left( \rho_{\text{DN}A}-\rho_{\text{water}} \right)V_{cyl}\frac{sin\left( q\frac{L}{2}\cos\frac{\alpha}{2} \right)}{q\frac{L}{2}\cos\frac{\alpha}{2}}\frac{2J_{1}\left( qR\sin\alpha\right)}{qR\sin\alpha}$ | (3.2) |

Here, $J_{1}$ is the first order Bessel function, $\alpha$ the angle between the cylinder axis and the ***q****-vector*, *L* the length of the cylinder core, *R* the radius of the cylinder core, $V_{\text{cyl}}$ is the cylinder volume and $\rho_{\text{DNA}/\text{water}}$ the electron density of DNA and water, respectively.

| $F_{\text{deb}}\left( q \right)=\frac{2\left( e^{-q^{2}R_{g}^{2}}-1+q^{2}R_{g}^{2} \right)}{\left( q^{2}R_{g}^{2} \right)^{2}}$ | (3.3) |
| --- | --- |
| $F_{\text{pow}}\left( q \right)=q^{-x}$ | (3.4) |

*R_g_* is the radius of gyration and *x* the exponent of the power law.

| $F_{\text{lor}}\left( q \right)=\frac{1}{1+\left( \frac{q-q_{0}}{B} \right)^{2}}$ | (3.5) |
| --- | --- |

Here, *q_0_* is the peak position and *B* is the half-width-half-maximum of the Lorentz peak. *q_0_* is directly related to the lattice spacing *d* via $d=\frac{2\pi}{q_{0}}$. For DNA origamis with a hexagonal lattice, i.e. a 24HB and the 4-LB, we have $a_{\text{hexagonal}}=\sqrt{\frac{4}{9}}d=\frac{2}{3}\frac{2\pi}{q_{0}}$ as a result of the relation $\frac{1}{d}=\frac{4}{3}\left( \frac{h^{2}+hk+k^{2}}{a^{2}} \right)$ which is valid for a 2D hexagonal lattice with *h* and *k* as Miller indices. For DNA origami with a square lattice structure, i.e. the 3-LB, the interhelical distance is calculated via $a_{\text{square}}=d=\frac{2\pi}{q_{0}}$.

To analyse the scattering data of 4-LB@silca a cuboid model as given in equation (3.6) and (3.7) is used instead of a cylinder model^2^.

| $F_{\text{cuboid}}\left( q,\alpha,\beta\right)=\int_{0}^{1} \phi_{q}\left( \mu\sqrt{1-\sigma^{2}},a \right)\left[ S\left( \frac{\mu c\sigma}{2} \right) \right]^{2}d\sigma$ | (3.6) |
| --- | --- |
| with $\phi_{q}\left( \mu,a \right)=\int_{0}^{1} \left\{ S\left[ \frac{\mu}{2}\cos\left( \frac{\pi}{2}u \right) \right]S\left[ \frac{\mu a}{2}\sin\left( \frac{\pi}{2}u \right) \right] \right\}^{2}du$, | (3.7) |

$S\left( x \right)=\frac{\sin\left( x \right)}{x}$ , and$\mu=qB$. The substitution of $\sigma=\cos\alpha$ and $\beta=\frac{\pi}{2}u$ are applied. Here, *A*, *B*, and *C* are the axis dimensions of the parallelepiped, $\Delta\rho$ the scattering contrast between DNA and water, $\alpha$ the angle between ***C*** and ***q****, and* $\beta$ the angle between the projection of the particles in the *xy-*plane and *y-*axis*.* Furthermore, it is assumed, that *a = A/B < 1,* *b = B/B = 1*, and *c = C/B > 1*.

During the model fitting the electron density of DNA $\rho_{\text{DNA}}$ and water $\rho_{\text{water}}$, the length of the 24HB *L*, and the widths *B* and *C* of 4-LB were fixed to $\rho_{DNA}=13\cdot{10}^{-6}Å^{-2}$, $\rho_{\text{water}}=9.4\cdot{10}^{-6}Å^{-2}$,$\rho_{\text{SiO2}}=19\cdot{10}^{-6}Å^{-2}$, $L=1000Å$, $B^{\text{4-LB}}=379Å$, $C^{\text{4-LB}}=555Å$, $B^{\text{3-LB}}=379Å$, and $C^{\text{3-LB}}=627Å$. Model fitting was achieved by running the software internal population-based DREAM algorithm using the software package SasView (SasView, 2014). The *q*-range dominated by aggregation is excluded from model fitting.

**4: Calculation of the Porod invariant**

The Porod invariant $Q$ is a model-free measure of the total scattering contrast. For a two-phase system it is calculated via

$Q=\int_{0}^{\infty} I\left( q \right)q^{2}dq=2\pi^{2}\phi_{b}\left( 1-\phi_{b} \right){\Delta\rho}^{2}$. (4.1)

Here, the volume fraction of the silicified DNA origami is called $\phi_{b}$and the water fraction is therefore given by $\left( 1-\phi_{b} \right)$. $\Delta\rho$ denotes the scattering contrast between the water and the silicified DNA origami. Thus, $Q$ provides a measure of the volume fraction and the total scattering contrast, which is in our case the dominant contribution. Thus, monitoring $Q$ allows to trace the silica growth. The calculation of $Q$ relies on the extrapolation of the experimental scattering data $I\left( q \right)$ to small and large *q.* For extrapolation to small *q*, we fit the data to the Guinier function ${I\left( q \right)=I}_{0}\cdot e^{\frac{-q^{2}R_{g}^{2}}{3}}$. Here, $R_{g}$ denotes the radius of gyration, which quantifies the objects distribution of scattering length density. We further omit extrapolation of the data to large q due to increased noise and limit the integration to $q_{\text{max}}=0.35Å^{-1}$. This way, we obtain the Porod invariant Q as function of silicification time^3, 4^.

**5: Contrast matching**

The concept of contrast matching, i.e. the vanishing of the scattering contrast between the DNA helices and their surrounding matrix, can be utilized to estimate the corresponding silica volume fraction.

| $x_{\text{SiO}_{2}}\cdot\rho_{\text{SiO}_{2}}+x_{\text{H}_{\text{2}}\text{O}}\cdot\rho_{\text{H}_{\text{2}}\text{O}}=\rho_{\text{DNA}}$ | (5.1) |
| --- | --- |

For the contrast matching condition, the volume fraction weighted electron density of the water-silica mixture with the respective electron densities $\rho_{\text{H}_{\text{2}}\text{O}}$ and $\rho_{\text{SiO}_{2}}$ is equal to the electron density of DNA $\rho_{DNA}$ (cf. Equation (1)). Here, $x_{\text{SiO}_{2}}$denotes the volume fraction of silica and $x_{H_{2}O}$ denotes the volume fraction water. With $x_{\text{H}_{\text{2}}\text{O}}=1-x_{\text{SiO}_{2}}$, $\rho_{\text{DNA}}=13\cdot{10}^{-6}Å^{-2}$, $\rho_{\text{water}}=9.4\cdot{10}^{-6}Å^{-2}$, and $\rho_{\text{SiO2}}=19\cdot{10}^{-6}Å^{-2}$ Equation (1) can be solved for $x_{\text{SiO}_{2}}$.

For an estimation of the DNA volume fraction of bare 24HBs, the standard atomic DNA volume in the 24HB (p8064 scaffold) is calculated via

| $1760\cdot V_{\text{Guanine}}+1767\cdot V_{\text{Cytosine}}+1942\cdot V_{\text{Adenine}}+2595\cdot V_{\text{Thymine}}$ | (5.2) |
| --- | --- |

to $2.47\cdot{10}^{6}Å^{3}$ ^5^ and compared to the cylinder volume obtained from SAXS measurements $V_{Cyl}=\pi R_{\text{bare}}^{2}L=20.2\cdot{10}^{6}Å^{3}$. This reveals 12 % DNA and 88 % water volume fraction for a bare 24HBs.

**6: Silicification of 24HBs – Parameter overview**

|  | *Q*  [${10}^{-3}{cm}^{-1}Å^{-3}$] | *R*  [Å] | *a*  [Å] | *t*  [h] |
| --- | --- | --- | --- | --- |
| Bare 24HBs | $Q_{\text{bare}}=0.3$ | $R_{\text{bare}}=80.1\pm0.2$ | $a_{\text{bare}}=26.2\pm0.3$ | t = 0 h |
| Most condensed 24HB@SiO_2_ |  | $R_{\text{min}}^{\text{SiO2}}=74.2\pm0.5$ | $a_{\text{min}}^{\text{SiO2}}=23.8\pm0.2$ | t = 4-8 h |
| Most condensed 24HB@TMAPS |  | $R_{\text{min}}^{\text{TMAPS}}=73.4\pm0.4$ | $a_{\text{min}}^{\text{TMAPS}}=25.2\pm0.3$ | t = 8 h |
| 24HB@SiO_2_ | $Q_{\text{SiO2}}=1.1$ | $R_{\text{SiO2}}=80.4\pm0.1$ | $a_{\text{SiO2}}=24.7\pm0.05$ | t >12 h |

**Supplementary Table *2*** The Porod invariant (*Q*), the cylinder Radius (*R*), the interhelical spacing (*a*) and the time (*t*) for bare, most condensed, TMAPS-only exposed, and silicified 24HBs. The parameters correspond to the data shown in Figure 1bc and 2 and were obtained from Porod invariant analysis as well as model fitting of the in-situ SAXS data.

**7: Silicification of 4-LBs – Parameter overview**

|  | *Q*  [${10}^{-3}{cm}^{-1}Å^{-3}$] | *A*  [Å] | *t*  [h] |
| --- | --- | --- | --- |
| Bare 4-LBs | $Q_{\text{bare}}=0.3$ | $A_{\text{bare}}=89.9\pm0.4$ | *t* = 0 h |
| Most condensed  4-LBs@SiO_2_ | $Q_{\text{SiO2}}=0.45$ | $A_{\text{min}}^{\text{SiO2}}=80.3\pm1.3$ | *t* = 56 h |

**Supplementary Table 3** The Porod invariant (Q), the cuboid height (A), and the time (t) for bare and silicified 4-LBs. The parameters correspond to the data shown in Figure 4bc and were obtained from Porod invariant analysis as well as model fitting of the in-situ SAXS data.

**8: Silicification of 3-LBs – Parameter overview**

|  | *Q*  [${10}^{-3}{cm}^{-1}Å^{-3}$] | *R*  [Å] | *t*  [h] |
| --- | --- | --- | --- |
| Bare 3-LBs | $Q_{\text{bare}}=0.35$ | $A_{\text{bare}}=80.0\pm0.4$ | *t* = 0 h |
| Most condensed  3-LBs @SiO_2_ |  | $A_{\text{min}}^{\text{SiO}\text{2}}=62.8\pm1.7$ | *t* = 2 h |
| Most condensed  3-LBs@TMAPS |  | $A_{\text{min}}^{\text{TMAPS}}=55.5\pm1.1$ | *t* > 6 h |
| 3-LBs@SiO_2_ | $Q_{\text{SiO2}}=2.2$ | $A_{\text{SiO2}}=109.7\pm0.7$ | *t* >48 h |

**Supplementary Table 4** The Porod invariant (Q), the cuboid height (A), and the time (t) for bare, most condensed, TMAPS-only exposed, and silicified 3-LBs. The parameters correspond to the data shown in Supplementary Figure 10bc and 11b and were obtained from Porod invariant analysis as well as model fitting of the in-situ SAXS data.

# **9: In situ silicification of 24HBs monitored by SAXS – Lorentzian peaks**


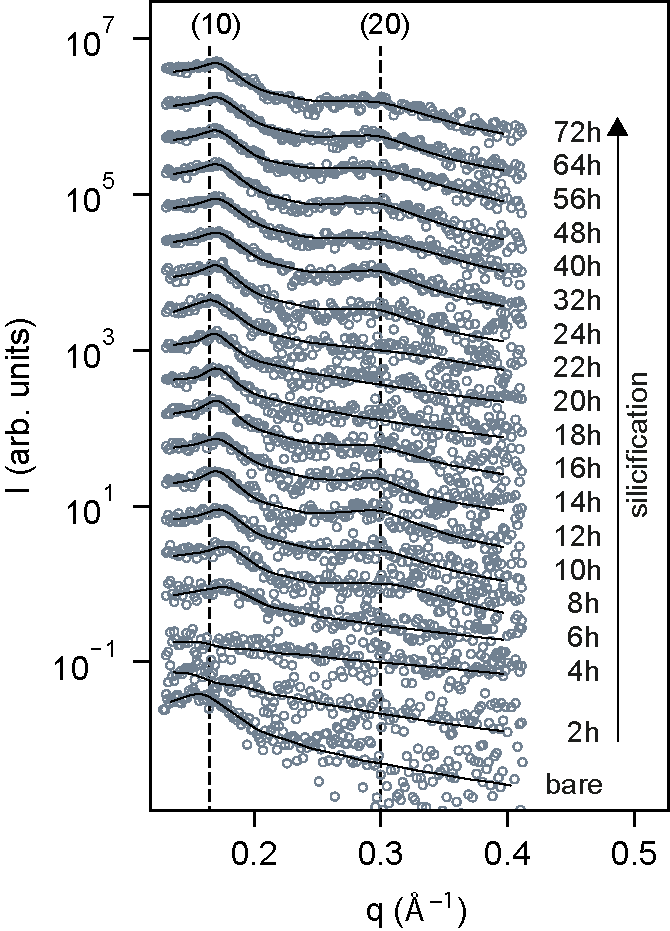


**Supplementary Figure 5** In situ silicification of 24HBs (main text Figure 1a). Enlarged view of the q-range sensitive to the inner lattice design. SAXS intensities are shown together the best fits of a cylinder model together with Lorentzian peaks accounting for the inner honeycomb lattice arrangement. The first order (10) and second order (20) honeycomb lattice peaks are highlighted by dashed lines. Data is scaled for clarity.

**10: SAXS data of 24HBs exposed to TMAPS only for several hours**


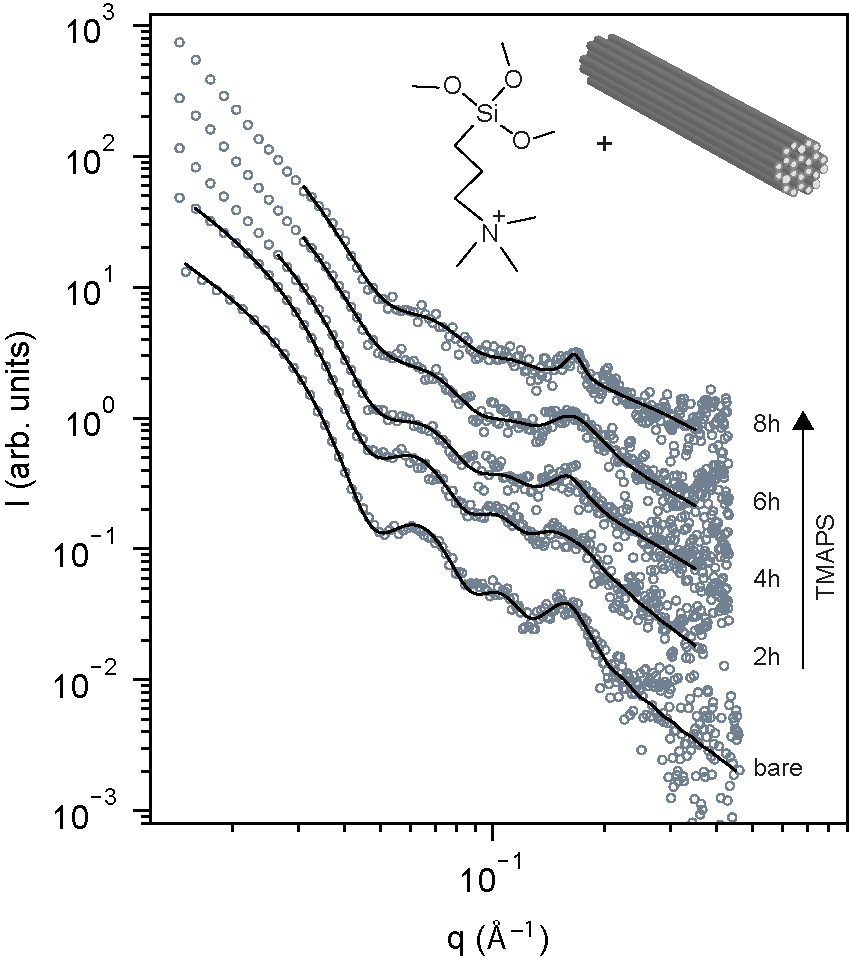


**Supplementary Figure 6** SAXS intensities of bare 24HBs and after the addition of TMAPS together with the best fits of a cylinder model and Lorentz peaks accounting for the inner honeycomb lattice arrangement. Data is scaled for clarity.

It is noteworthy to mention here that contrast matching does not occur in the “TMAPS-only” case, as there is no formation of larger silica networks due to the absence of TEOS.

**11: Temperature stability of ultrathin silica-coated 4-LBs**

The thermal stability of condensed ultrathin silica-coated 4-LBs@SiO_2_ ($A_{\text{SiO2}}=86.2\pm1.7$ $Å$) is demonstrated by heating the structures to 60 °C for 30 min and subsequent SAXS analysis. Uncoated origami completely dissolve at this temperature^1^, whereas the origami@SiO_2_ remain stable.


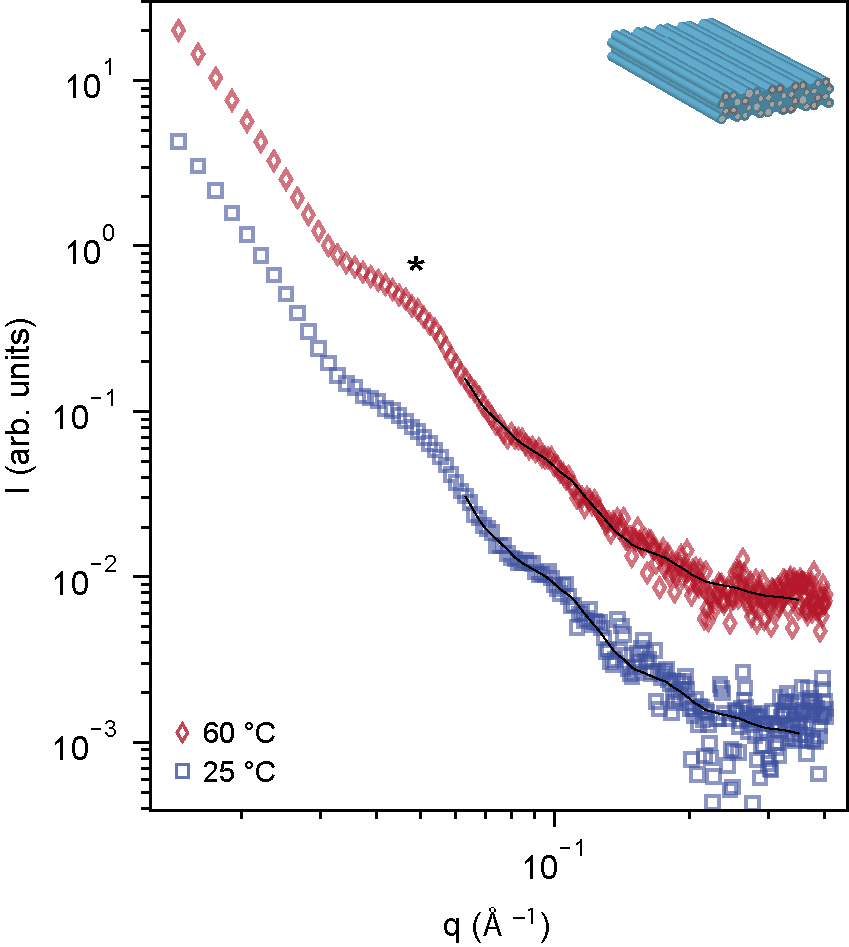


**Supplementary Figure 7** SAXS intensities of condensed ultrathin silica-coated 4-LBs@SiO_2_ measured at room temperature (blue squares) and after heating the structures to 60 °C for 30 min (red diamonds). The 4-LB@SiO_2_ stacking peak visible in the low q-regime is highlighted. Centre-to-centre distance is calculated to 137± 3 Å.


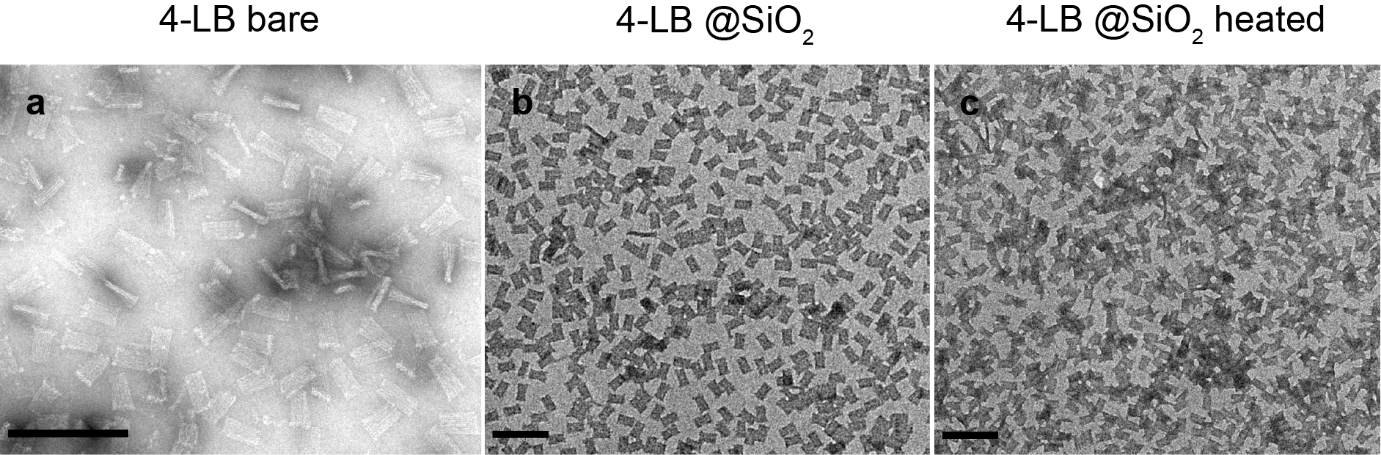


**Supplementary Figure 8** Temperature stability of condensed ultrathin silica-coated 4-LBs @SiO_2_ verified by TEM. TEM micrograph of (a) bare 4-LBs, (b) 4-LB @SiO_2_ at room temperature and (c) after heating to 60 °C for 30 min are shown. Scale bars: 200 nm.

**12: TEM images of aggregated silicified 4-LBs**


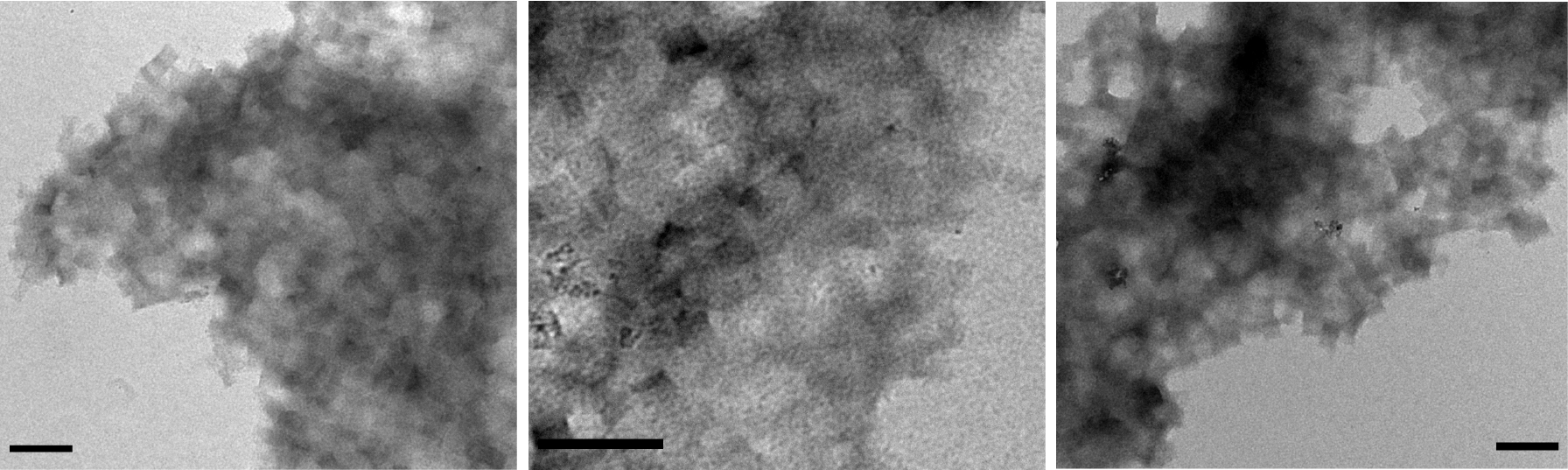


**Supplementary Figure 9** Aggregated 4-LBs after silicification for > 24h with a nucleotide: TMAPS: TEOS ratio of 1:5:20. Scale bars are 200 nm.

# **13: In-situ silicification of 3-LBs with a square lattice design**

**Materials and Methods**

Folding and purification of DNA origami structures

The DNA origami structures used here was designed using the CaDNAno software^6^ (design schematics in Supplementary Figure 1 and Supplementary Table 1).

3-LB*:* The *3-LB* structure (design schematics in Supplementary Figure 1c) was folded using 30 nM of DNA scaffold p8064 (tilibit nanosystems GmbH, Germany), and 100 nM of each staple oligonucleotide (Eurofins Genomics Germany GmbH and Integrated DNA Technologies, Inc., USA) in buffer containing 400 mM Tris-Acetate, 1 mM EDTA (pH = 8) and 14 mM MgCl_2_. The mixture was heated to 65 °C and held at this temperature for 15 min, then slowly cooled down to 4 °C over a period of 15 h^1^.

The 3-LBs were concentrated and purified from excess staples by two rounds of polyethylene glycol (PEG) precipitation and re-dispersion in buffer (1x TE, 3 mM MgCl_2_)^1^. Concentration of the purified DNA origami solution (up to 270 nM or 1.4 g/L) was verified via absorption measurements (Thermo Scientific NanoDrop 1,000 Spectrophotometer). The successful folding of structures was confirmed by TEM analysis. DNA origami solutions were stored at 4 °C until further use.

Silica coating

For silicification of 3-LBs we followed the same protocol as for 4-LBs described in detail in the methods section of the main text.

We observe silicification-induced condensation not only for DNA origami structures with an inner honeycomb lattice arrangement, i.e. 24HBs and 4-LBs, but also for a second type of origami structure, i.e. a cuboid-shaped three-layer block (3-LB), which consists of 42 DNA double helices ordered on a square lattice (cf. Supplementary Figure 10). Similar to the 24HBs and the 4-LBs, we find a quickly increasing Porod invariant Q after the addition of TEOS as function of time (Supplementary Figure 10b). The 3-LB height undergoes, equivalent to the cylinder radius of the 24HBs, condensation at first, followed by a substantial expansion of 3-LBs' height (Supplementary Figure 10c). A minimal value of the cuboid height is reached after ~ 2 h with $A_{min}^{3-LB}=62.8\pm1.7Å$.


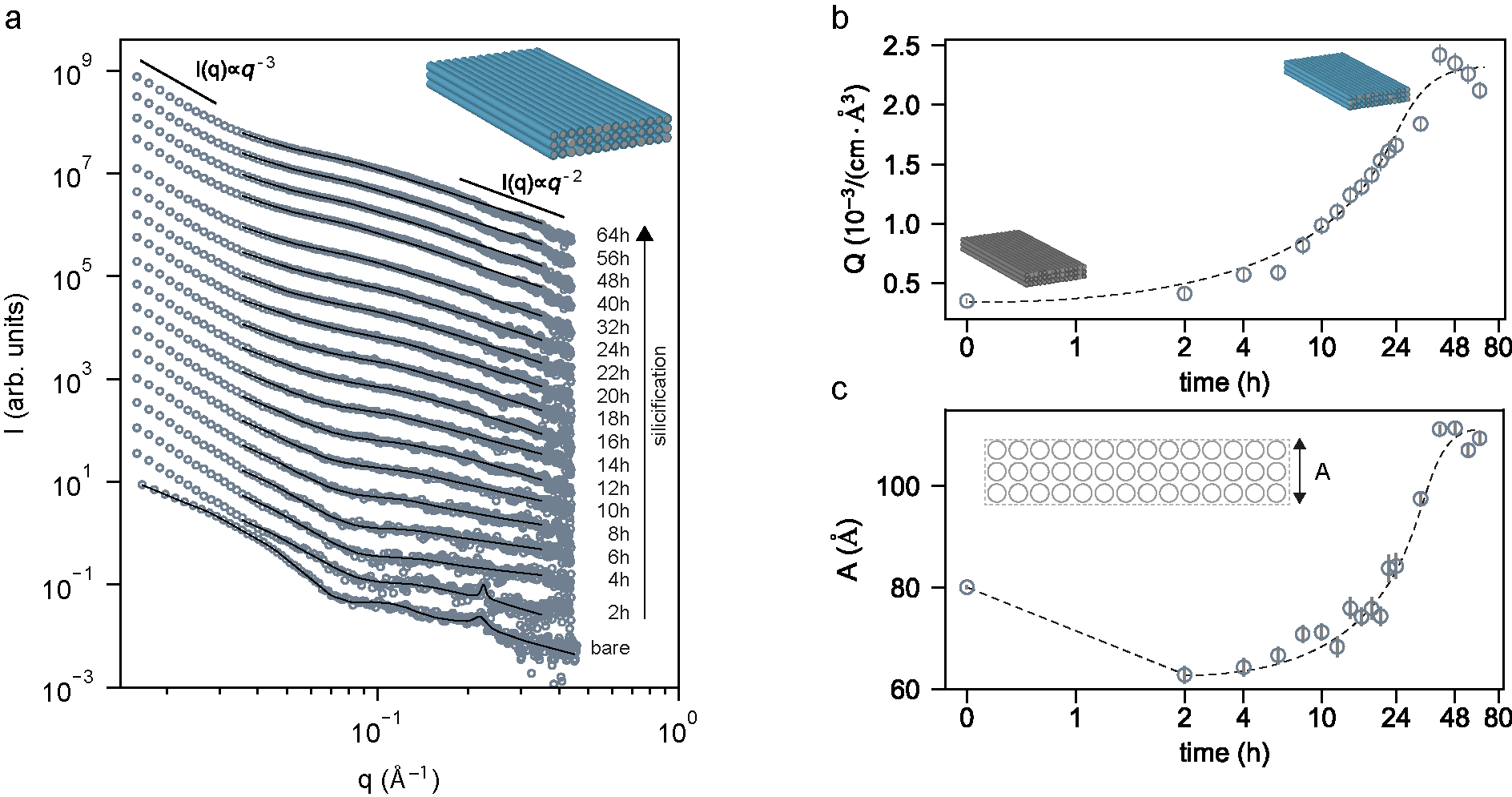


**Supplementary Figure 10** In-situ silicification of 3-LBs. (a) SAXS data recorded for bare 3-LBs and during silicification. SAXS intensities are shown together with the best fits of a cuboid model and Lorentzian shaped peaks accounting for the inner square lattice arrangement. Data is scaled for clarity. (b) Model-free Porod invariant Q calculated for the data shown in (a) as function of silica growth time. Error bars indicate standard deviation σ induced by counting statistics of the x-ray data. (c) Heights of the cuboid-shaped 3-LBs as function of silica growth time. Dashed lines serve as guide to the eye. Error bars indicate standard deviation σ due to modelling of the x-ray data for each time point.

After 48h *Q* reaches a plateau at $Q_{\text{SiO2}}^{\text{3-LB}}\left( t>48h \right)=2.2\cdot{10}^{-3}{cm}^{-1}Å^{-3}$ and the cuboid height calculates to $A_{\text{SiO2}}^{\text{3-LB}}=109.7\pm0.7Å$. Thus, we observe a particular large increase of the Porod invariant and the cuboid height in response to the silica growth on and within the 3-LBs, i.e. a large amount of silica deposition. Such thick silica encapsulation is in general likely accompanied by aggregation. Indeed, the SAXS intensities recorded during the silicification are quickly dominated by two power-laws as highlighted in Supplementary Figure 10a. This is consistent with the formation of fractal aggregates for which a power-law observable at low q-regions ($I\left( q \right)\propto q^{-3}$ corresponds to the scattering from the aggregates and the scattering of the particle surface manifests itself in a power-law at high q-values ($I\left( q \right)\propto q^{-2}$)^7^.

# **14: The influence of TMAPS on the 3-LBs**


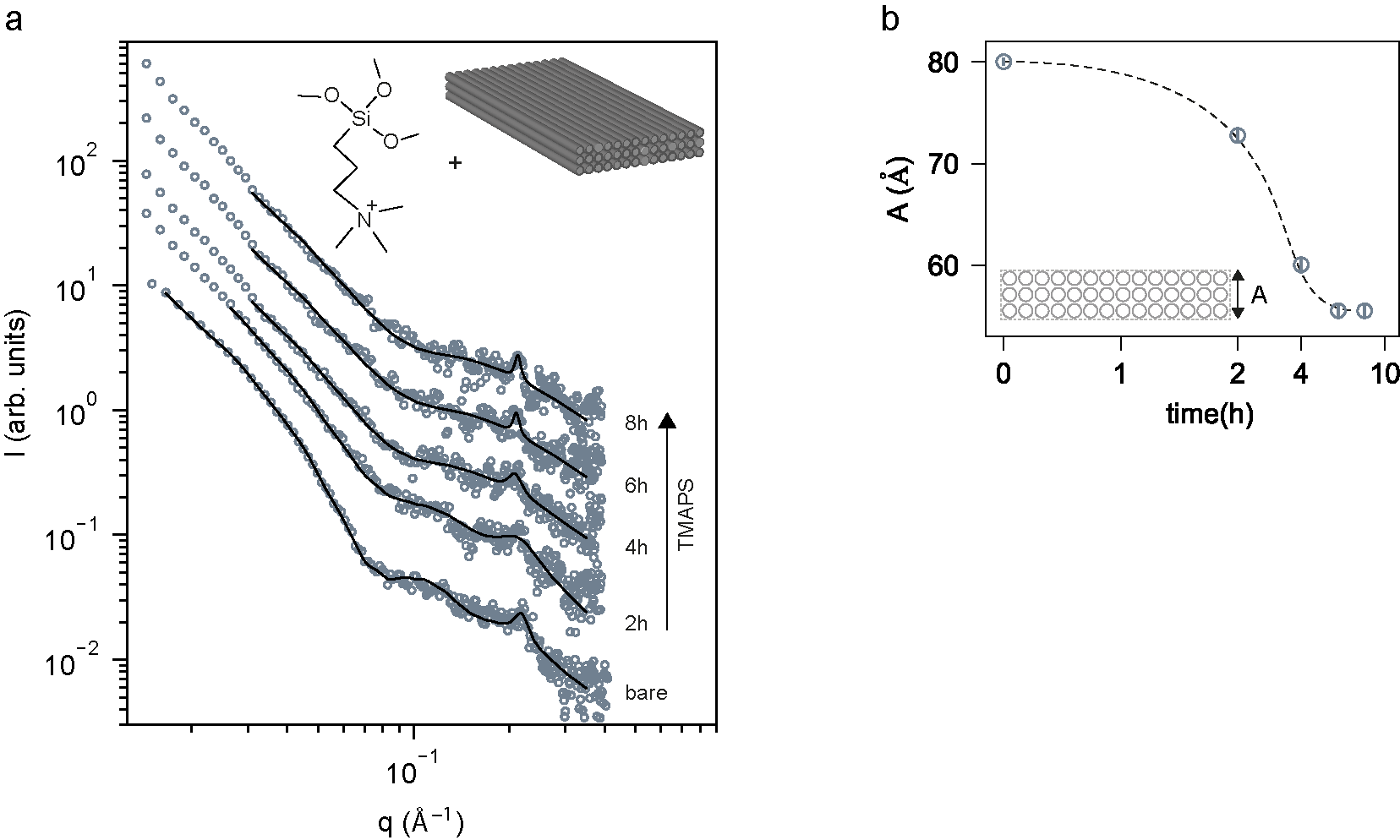


**Supplementary Figure 11** **(**a) SAXS intensities of bare 3-LBs and after the addition of TMAPS together with the best fits of a cuboid model and Lorentz peaks accounting for the inner lattice structure. Data is scaled for clarity. (b) Heights of the cuboid 3-LBs extracted from (a) as function of TMAPS incubation time. Error bars indicate standard deviation σ due to modelling of the x-ray data for each time point.

# **15: Estimation of TMAPS-TEOS primary particle size**

The length of a fully stretched out silica particle with different numbers of TEOS molecules was estimated taking into account the relevant bond lengths and bond angles from literature. The values given in Supplementary Table 5 represent the upper limit of the possible size of the primary particles. Since the primary silica particle is unlikely to be present in its fully stretched out state and branching of the silica particles must be taken into consideration, the actual values for the corresponding outer shell thicknesses will be significantly below this upper limit and are therefore in good agreement with the values obtained from the SAXS measurements.


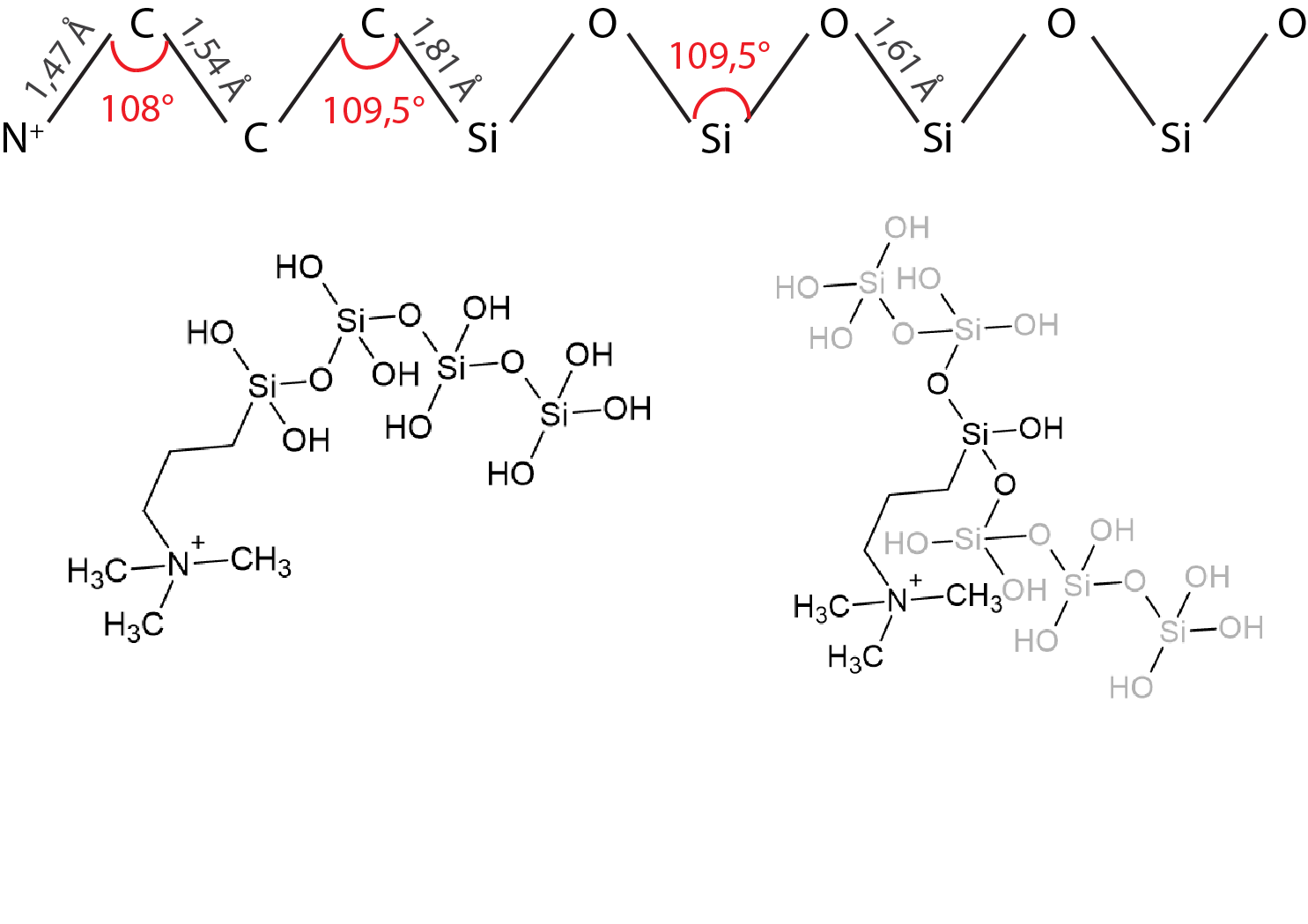


**Supplementary Figure 12** Upper image: Schematic minimal simplistic representation of a linear silica chain with four units including the bond lengths and bond angles used for a rough estimation of the length of such a fully stretched silica chain. Lower image(s): Molecular schemes of liner primary silica particles (left) and primary particles showing branching (right, different options for attached units are shown in grey).

| **Number of silica chain units** | **Estimated length of fully stretched silica chain** | **Estimation based on the ‘freely rotating chain’ model** |
| --- | --- | --- |
| 1 (TMAPS only) | 6.5 Å | 5.1 Å |
| 2 (TMAPS + 1 TEOS) | 9.1 Å | 6.0 Å |
| 3 (TMAPS + 2 TEOS) | 11.7 Å | 6.8 Å |
| 4 (TMAPS + 3 TEOS) | 14.3 Å | 7.5 Å |

**Supplementary Table 5** Table summarizing the estimated length for fully stretched silica chains and the estimated end-to-end distance based on the freely rotating chain model for different numbers of chain units.

**References**

1. Fischer S, Hartl C, Frank K, Radler JO, Liedl T, Nickel B. Shape and interhelical spacing of DNA origami nanostructures studied by small-angle X-ray scattering. *Nano Lett.* **16**, 4282-4287 (2016).

2. Mittelbach P, Porod G. Zur Röntgenkleinwinkelstreuung verdünnter kolloider Systeme. Die Berechnung der Streukurven von Parallelepipeden. *Acta Phys. Austriaca.* **14**, 185-211 (1961).

3. Glatter O, Kratky O. Small angle X-ray scattering. *Academic Press Inc. Ltd.* London (1982).

4. Mantella V*, et al.* Polymer lamellae as reaction intermediates in the formation of copper nanospheres as evidenced by in situ X-ray studies. *Angew. Chem. Int. Ed.* **59**, 11627–11633 (2020).

5. Nadassy K, Tomás-Oliveira I, Alberts I, Janin J, Wodak SJ. Standard atomic volumes in double-stranded DNA and packing in protein–DNA interfaces. *Nucleic Acids Res.* **29**, 3362 (2001).

6. Douglas SM, Dietz H, Liedl T, Högberg B, Graf F, Shih WM. Self-assembly of DNA into nanoscale three-dimensional shapes. *Nature* **459**, 414-418 (2009).

7. Rieker TP, Hindermann-Bischoff M, Ehrburger-Dolle F. Small-angle X-ray scattering study of the morphology of carbon black mass fractal aggregates in polymeric composites. *Langmuir* **16**, 5588-5592 (2000).
